# Supplementary material for: Multi-step nucleation pathway of C-S-H during cement hydration from atomistic simulations
Source: Nat Commun. 2023 Dec 2;14:7979. doi: 10.1038/s41467-023-43500-y (PMC10693585; doi:10.1038/s41467-023-43500-y)
Supplement: Supplementary file 1 — Supplementary Information [file 41467_2023_43500_MOESM1_ESM.pdf]

Supplementary information: Multi-step nucleation pathway of C-S-H during cement hydration from atomistic simulations

Xabier M. Aretxabaleta[1,*], Jon López-Zorrilla[1], Iñigo Etxebarria[1,2], and Hegoi Manzano[1,*]

[1]Fisika saila, Euskal Herriko Unibertsitatea UPV/EHU, Sarriena Auzoa z/g, 48940 Leioa, Basque Country, Spain
[2]EHU Quantum Center, Euskal Herriko Unibertsitatea, UPV/EHU, Spain
[*]xabier.mendez@ehu.eus

ABSTRACT

C-S-H nucleation is a crucial step during cement hydration and determines to a great extent the rheology, microstructure, and properties of the cement paste. Recent evidence indicates that the C-S-H nucleation involves at least two steps, yet the underlying atomic scale mechanism, the nature of the primary particles and their stability, or how they merge/aggregate to form larger structures is unknown. In this work, we use atomistic simulation methods, specifically DFT, evolutionary algorithms (EA), and Molecular Dynamics (MD), to investigate the structure and formation of C-S-H primary particles (PPs) from the ions in solution, and then discuss a possible formation pathway for the C-S-H nucleation. Our simulations indicate that even for small sizes the most stable clusters encode C-S-H structural motifs, and we identified a $C_4S_4H_2$ cluster candidate to be the C-S-H basic building block. We suggest a formation path in which small clusters formed by silicate dimers merge into large elongated aggregates. Upon dehydration, the C-S-H basic building blocks can be formed within the aggregates, and eventually crystallize.

Ca and Si speciation

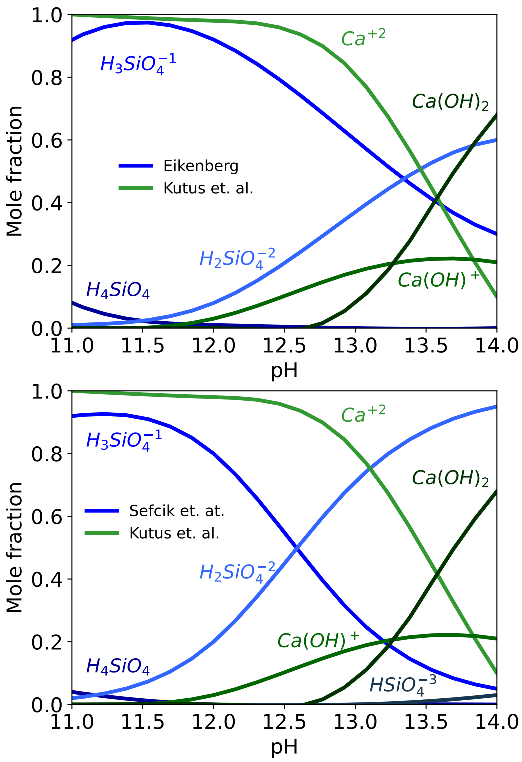

Figure S1. Ca and Si speciation in terms of pH from different sources. The data was obtained from ref[1–3].

Evolutionary Algorithms calculations energy evolution.

Figures S2-S10 show the energy evolution of all the individuals of every EA calculation. Pink dots represent structures created randomly, blue dots structures created by heredity operators, orange dots structures created by mutation operators and green dots structures that were kept for the next generation. The black line represents the lowest energy structure for every generation and the filled green curve represents the energy density of all the created structures.

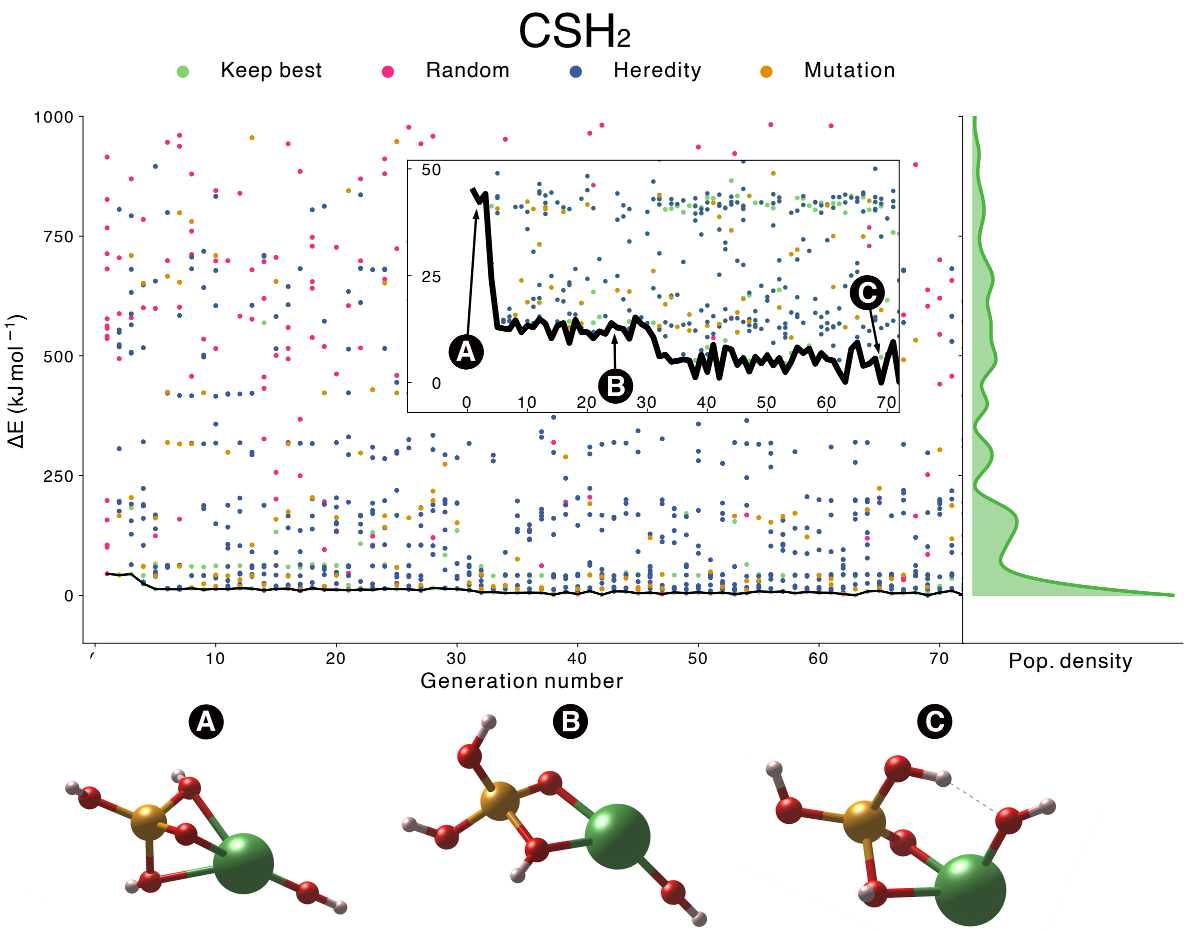

Figure S2. Energy evolution and energy population density of the Evolutionary Algorithms calculation for CSH_2 PPs. Each point represents a structure. Green points represent structures that has been kept from the previous generation. Pink points represent structures that have been created randomly. Blue points represent structures that have been created by using the heredity operator. Orange points represent structures that have been created using the mutation operator. The structures A, B, and C represent the best PP in generations 1,25 and 70.

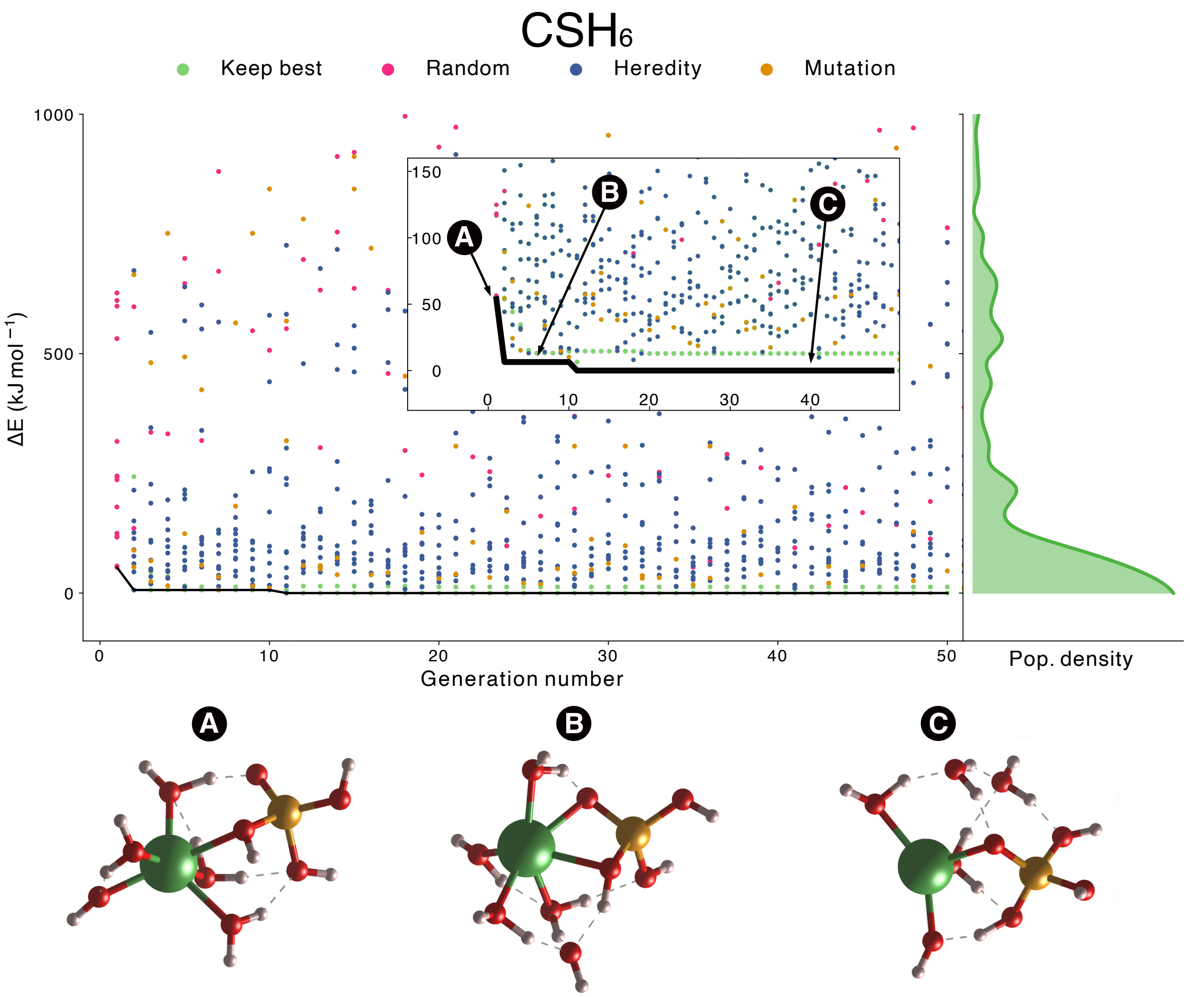

Figure S3. Energy evolution and energy population density of the Evolutionary Algorithms calculation for CSH_6 PPs. The structures A, B, and C represent the best PP in generations 1,5 and 40.

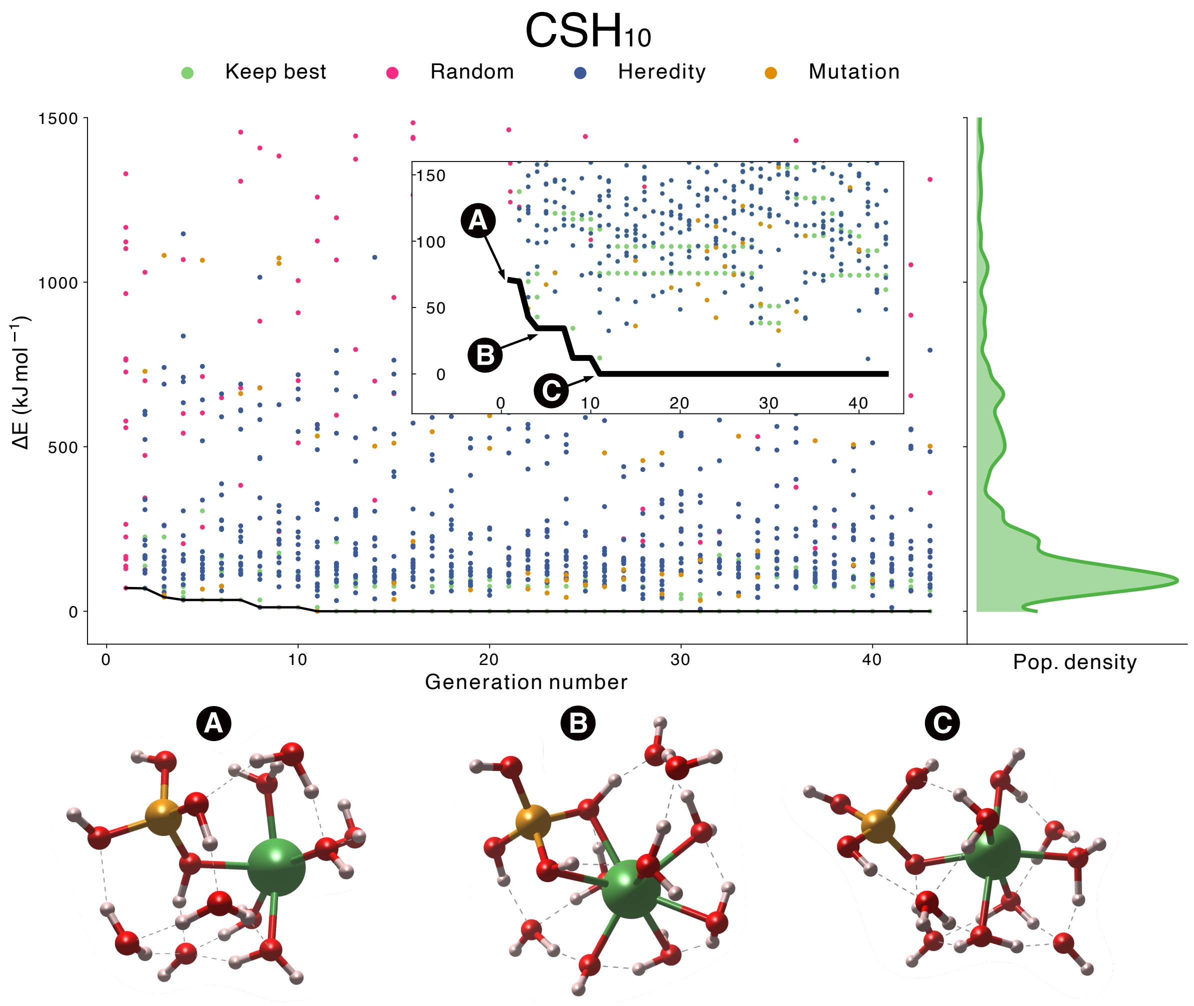

Figure S4. Energy evolution and energy population density of the Evolutionary Algorithms calculation for CSH_{10} PPs. Each point represents a structure. Green points represent structures that has been kept from the previous generation. Pink points represent structures that have been created randomly. Blue points represent structures that have been created by using the heredity operator. Orange points represent structures that have been created using the mutation operator. The structures A, B, and C represent the best PP in generations 1,5 and 12.

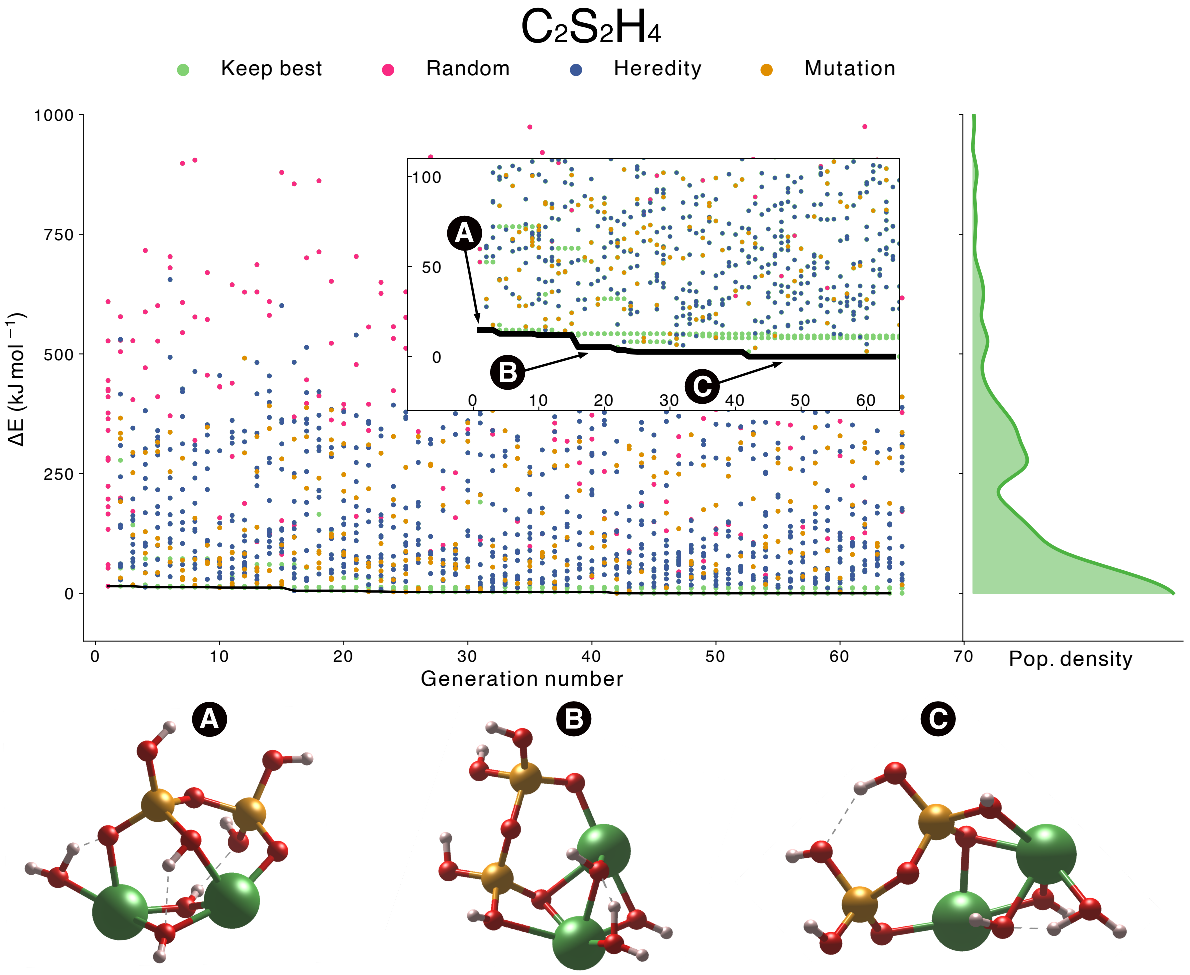

Figure S5. Energy evolution and energy population density of the Evolutionary Algorithms calculation for $C_2S_2H_4$ PPs. The structures A, B, and C represent the best PP in generations 1,20 and 50.

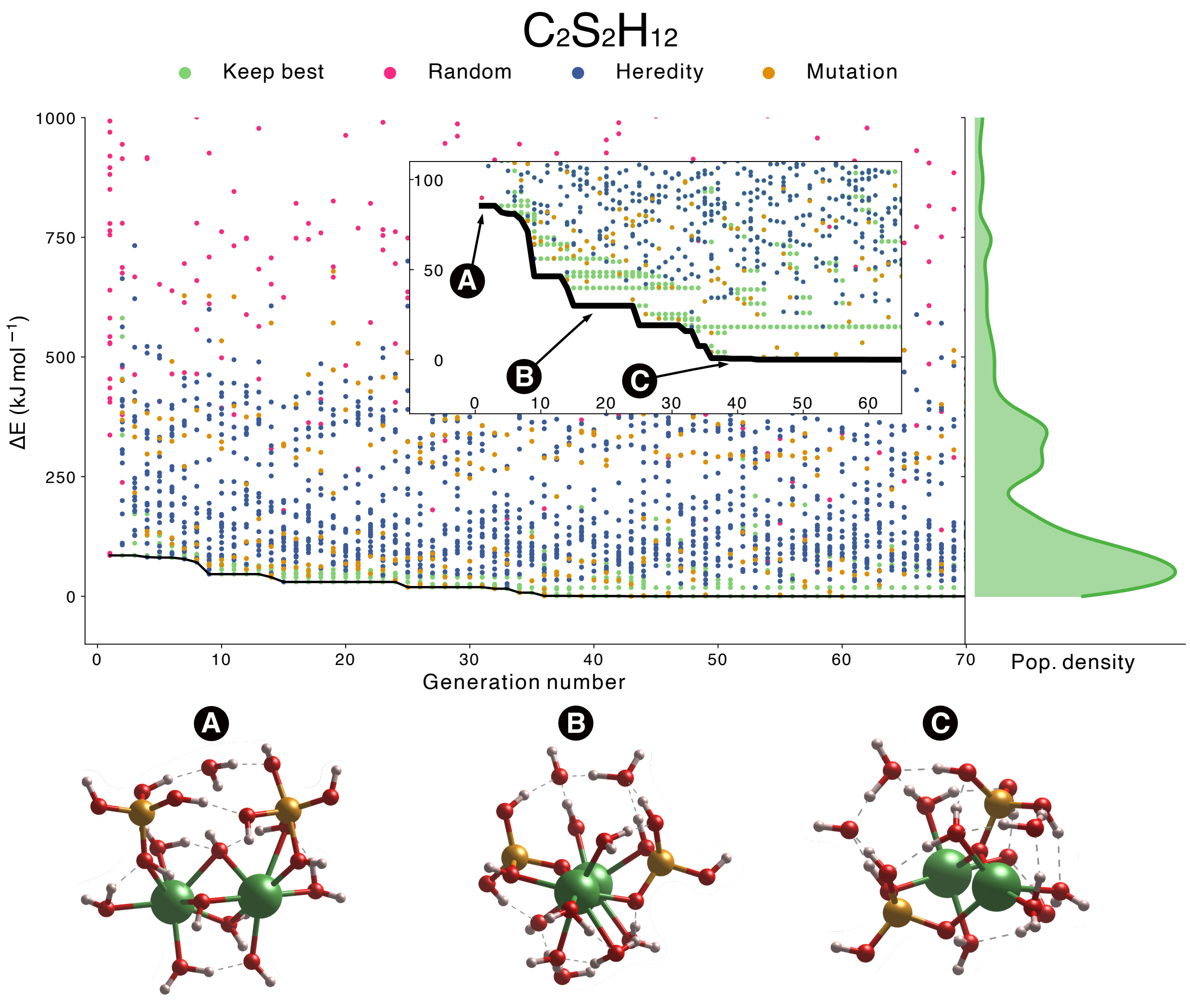

Figure S6. Energy evolution and energy population density of the Evolutionary Algorithms calculation for $C_2S_2H_{12}$ PPs. Each point represents a structure. Green points represent structures that has been kept from the previous generation. Pink points represent structures that have been created randomly. Blue points represent structures that have been created by using the heredity operator. Orange points represent structures that have been created using the mutation operator. The structures A, B, and C represent the best PP in generations 1,20 and 40.

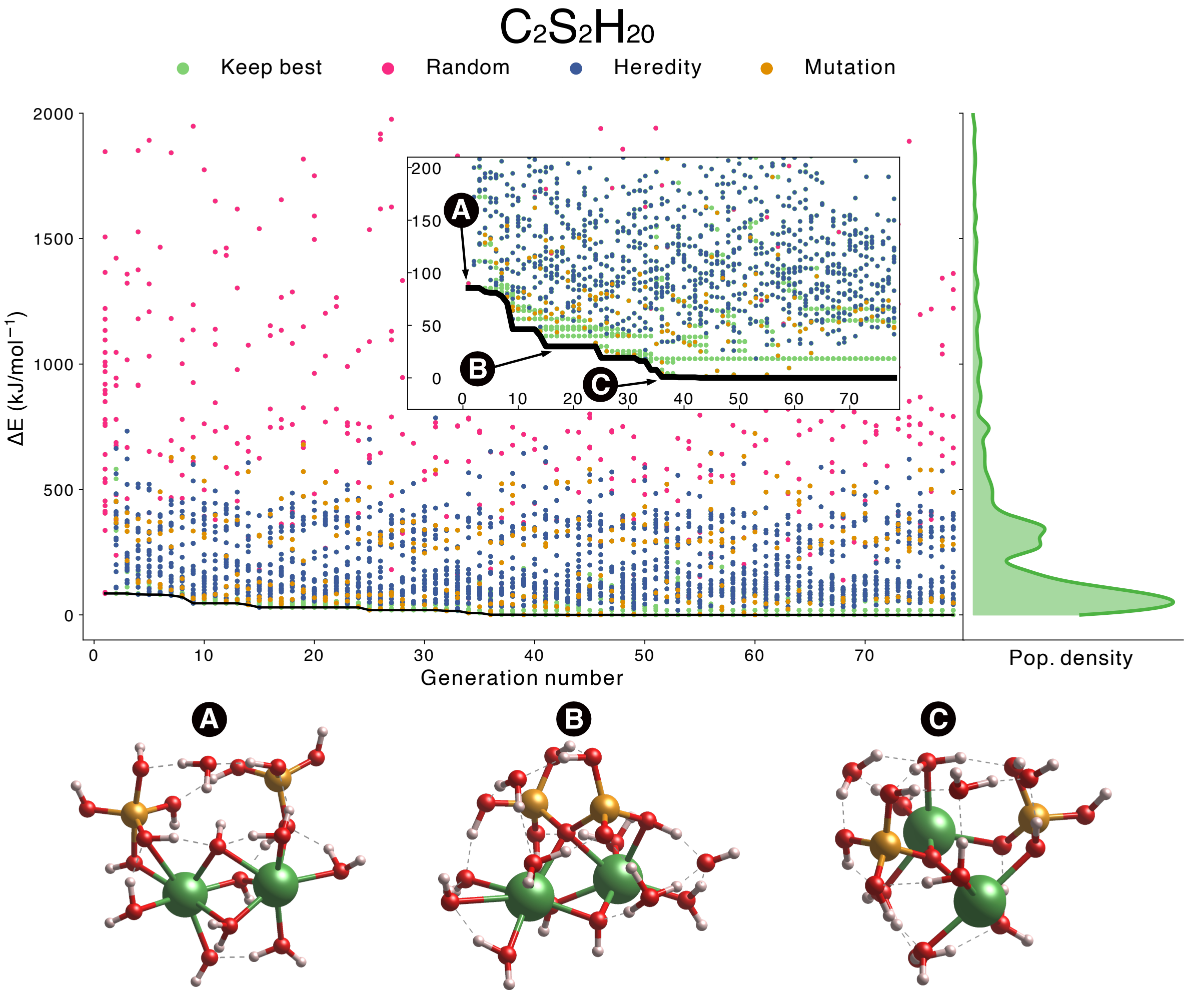

Figure S7. Energy evolution and energy population density of the Evolutionary Algorithms calculation for $C_2S_2H_{20}$ PPs. The structures A, B, and C represent the best PP in generations 1,20 and 40.

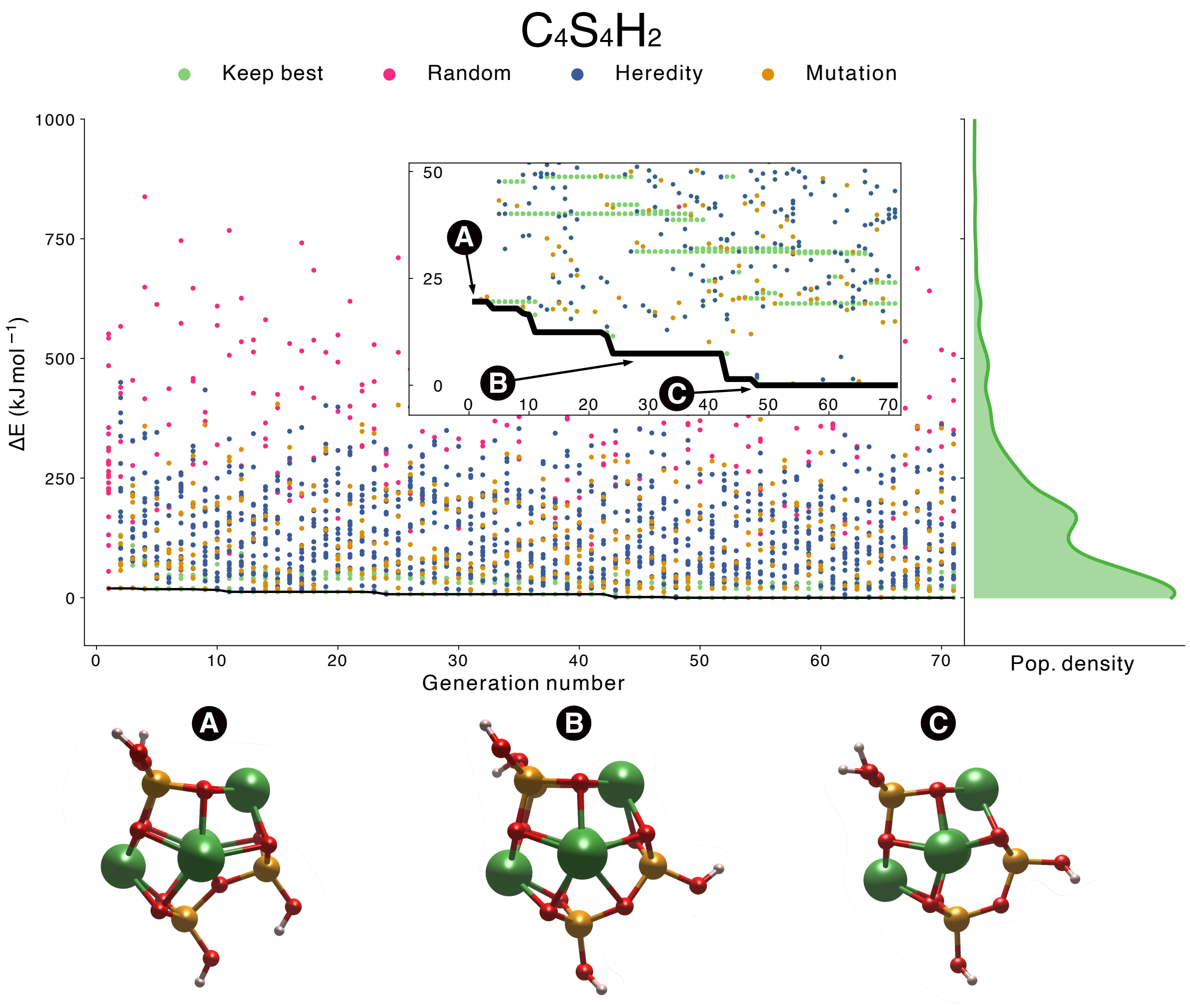

Figure S8. Energy evolution and energy population density of the Evolutionary Algorithms calculation for $C_4S_4H_2$ PPs. Each point represents a structure. Green points represent structures that has been kept from the previous generation. Pink points represent structures that have been created randomly. Blue points represent structures that have been created by using the heredity operator. Orange points represent structures that have been created using the mutation operator. The structures A, B, and C represent the best PP in generations 1,30 and 50.

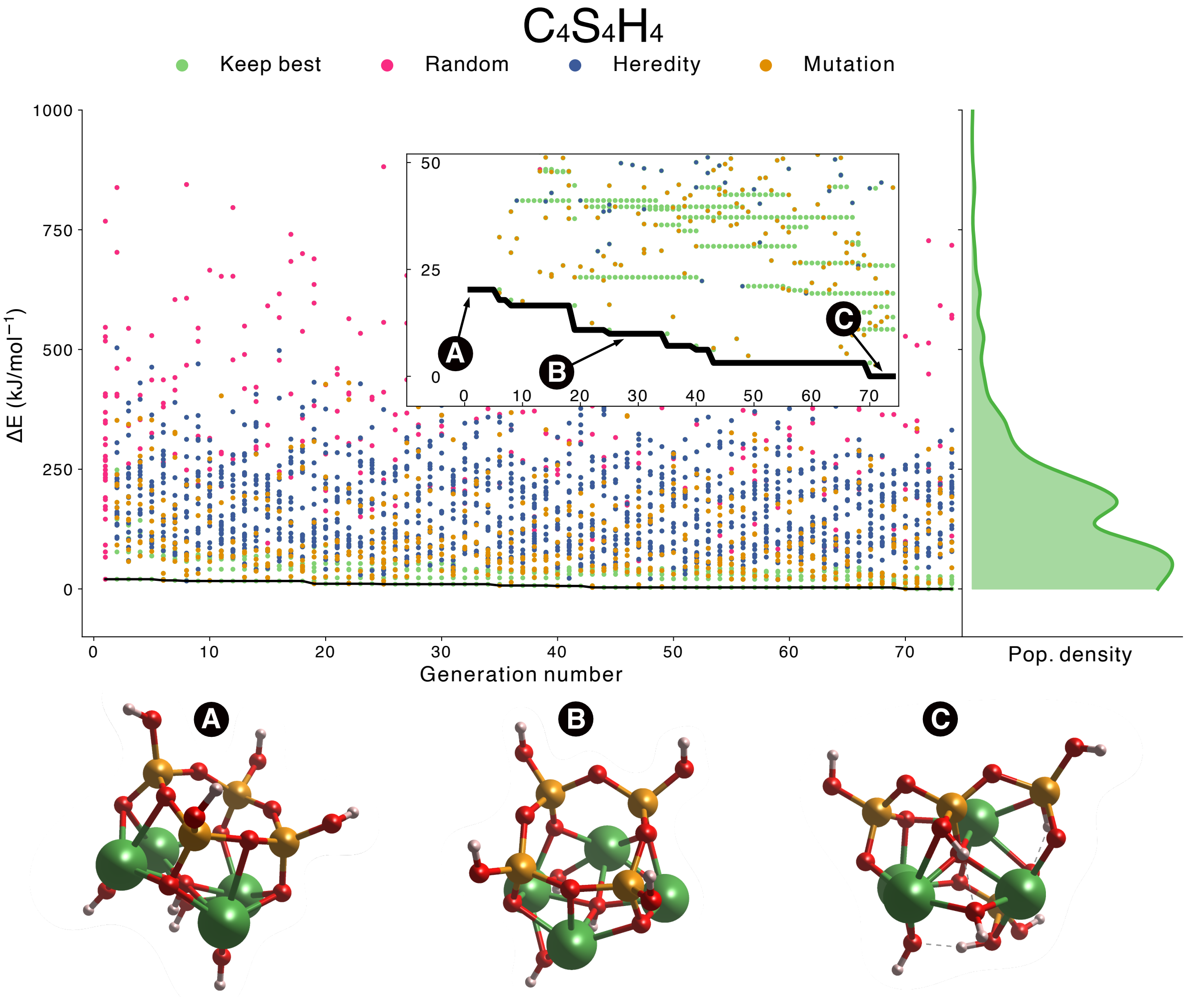

Figure S9. Energy evolution and energy population density of the Evolutionary Algorithms calculation for $C_4S_4H_4$ PPs. Each point represents a structure. Green points represent structures that has been kept from the previous generation. Pink points represent structures that have been created randomly. Blue points represent structures that have been created by using the heredity operator. Orange points represent structures that have been created using the mutation operator. The structures A, B, and C represent the best PP in generations 1,30 and 72.

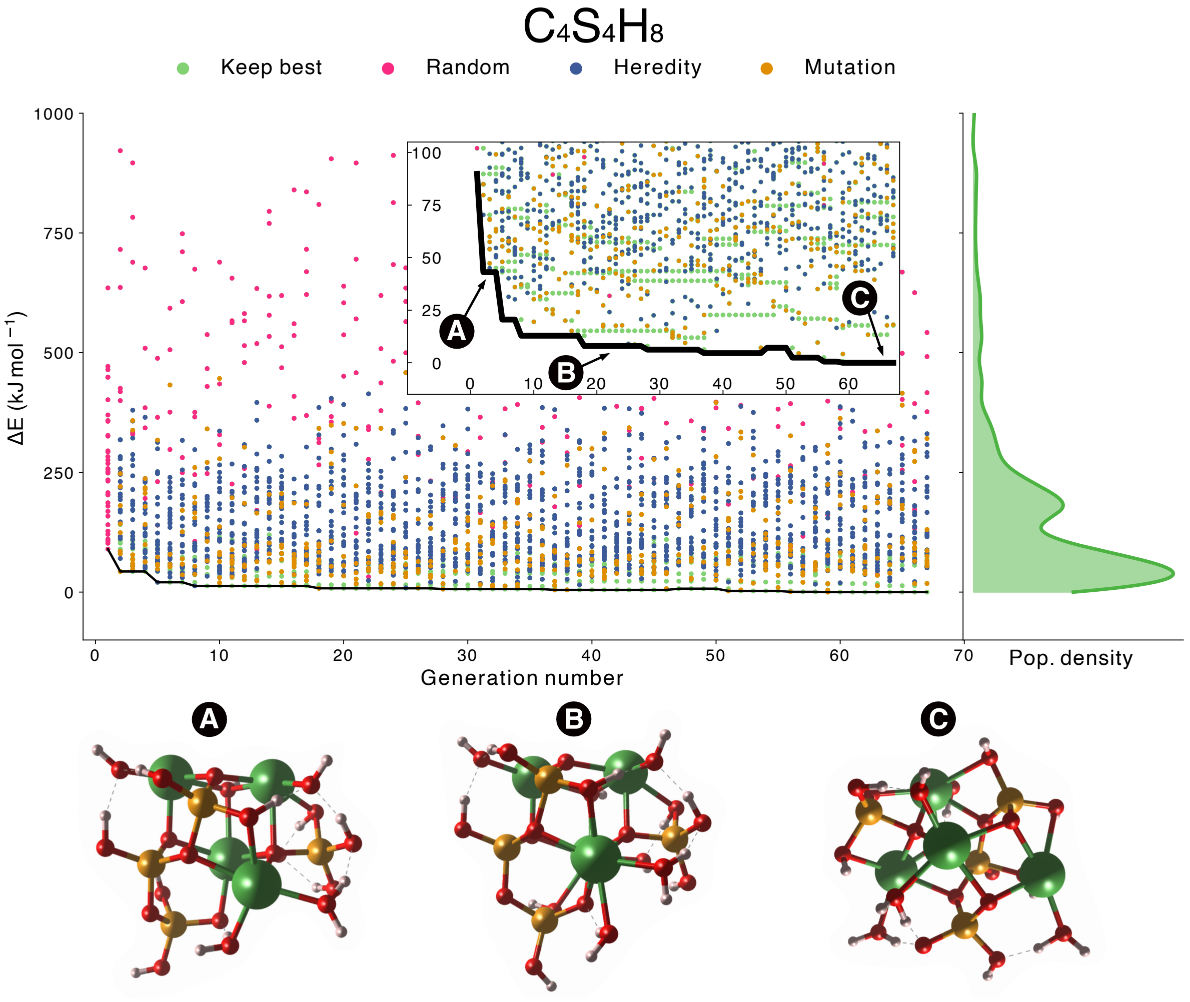

Figure S10. Energy evolution and energy population density of the Evolutionary Algorithms calculation for $C_4S_4H_8$ PPs. Each point represents a structure. Green points represent structures that has been kept from the previous generation. Pink points represent structures that have been created randomly. Blue points represent structures that have been created by using the heredity operator. Orange points represent structures that have been created using the mutation operator. The structures A, B, and C represent the best PP in generations 2,25 and 65.

Sketch-maps

Figures S11-S16 show the sketch-map of following stoichiometries: CSH_2, CSH_6, $C_2S_2H_4$, $C_2S_2H_{20}$, $C_4S_4H_2$ and $C_4S_4H_4$. These figures also shown different properties of the clusters by means of different colormaps. For a deeper analysis of the results, .json files are also included as supplementary material, ready to be used with the Chemiscope online tool http://www.chemiscope.org. The files are named as followed:

1. CSH_2 clusters → CSH2_chemiscope.json.gz
2. CSH_6 clusters → CSH6_chemiscope.json.gz
3. CSH_{10} clusters → CSH10_chemiscope.json.gz
4. $C_2S_2H_4$ clusters → C2S2H4_chemiscope.json.gz
5. $C_2S_2H_{12}$ clusters → C2S2H12_chemiscope.json.gz
6. $C_2S_2H_{20}$ clusters → C2S2H20_chemiscope.json.gz
7. $C_4S_4H_2$ clusters → C4S4H2_chemiscope.json.gz

8. $C_4S_4H_4$ clusters → C4S4H4_chemiscope.json.gz
9. $C_4S_4H_8$ clusters → C4S4H8_chemiscope.json.gz

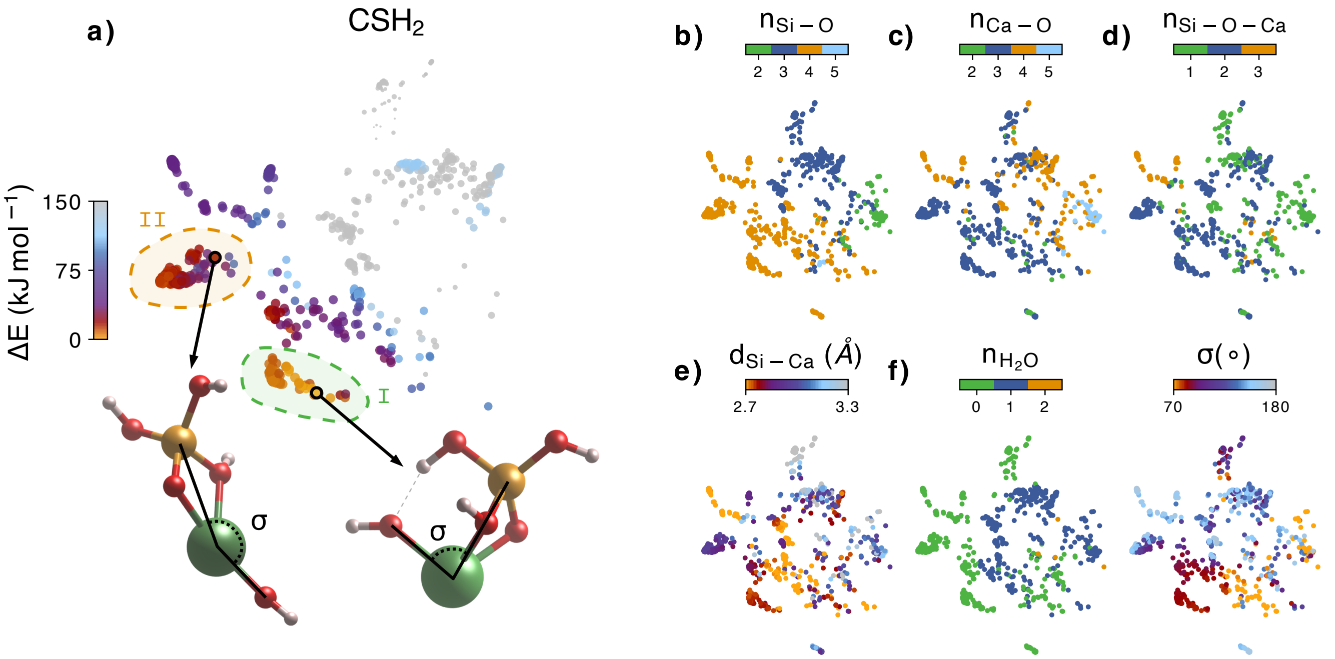

Figure S11. Sketch-map representation of the CSH_2 clusters. The colormap in **(a)** represents the energy difference ΔE. **(b-g)** Collection of structural properties of the CSH_2 clusters mapped into the same Sketch-map. n_{Si-O} refers to the number of Si-O bonds, n_{Ca-O} refers to the number of Ca-O bonds, $n_{Si-O-Ca}$ refers to the number of Si-O-Ca bonds, d_{Si-Ca} refers to the Si-Ca distance, n_{H_2O} refers to the number of water molecules and σ refers to the number of OH groups. Regions I and II each contain clusters with similar structural properties.

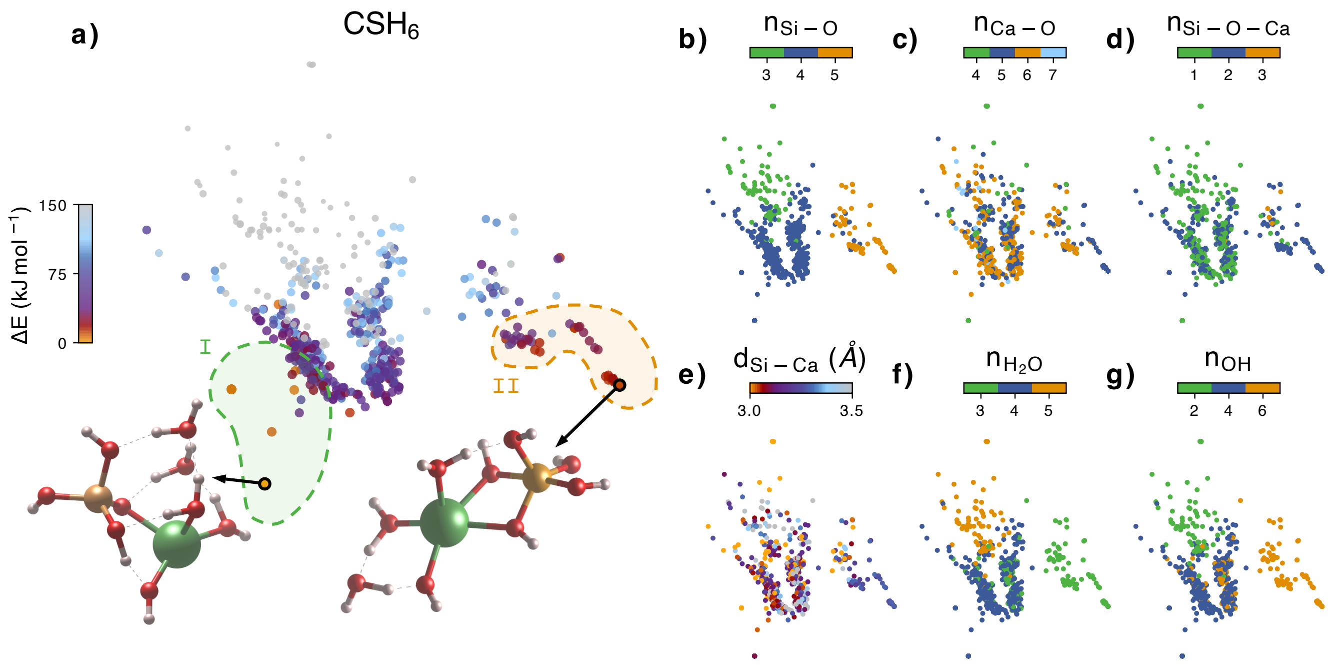

Figure S12. Sketch-map representation of the CSH_6 clusters. The colormap in **(a)** represents the energy difference ΔE. **(b-g)** Collection of structural properties of the CSH_6 clusters mapped into the same Sketch-map. n_{Si-O} refers to the number of Si-O bonds, n_{Ca-O} refers to the number of Ca-O bonds, $n_{Si-O-Sa}$ refers to the number of Si-O-Si bonds, $n_{Ca-O-Ca}$ refers to the Ca-O-Ca bonds, n_{H_2O} refers to the number of water molecules and n_{OH} refers to the number of OH groups. Regions I and II each contain one clusters with similar structural properties.

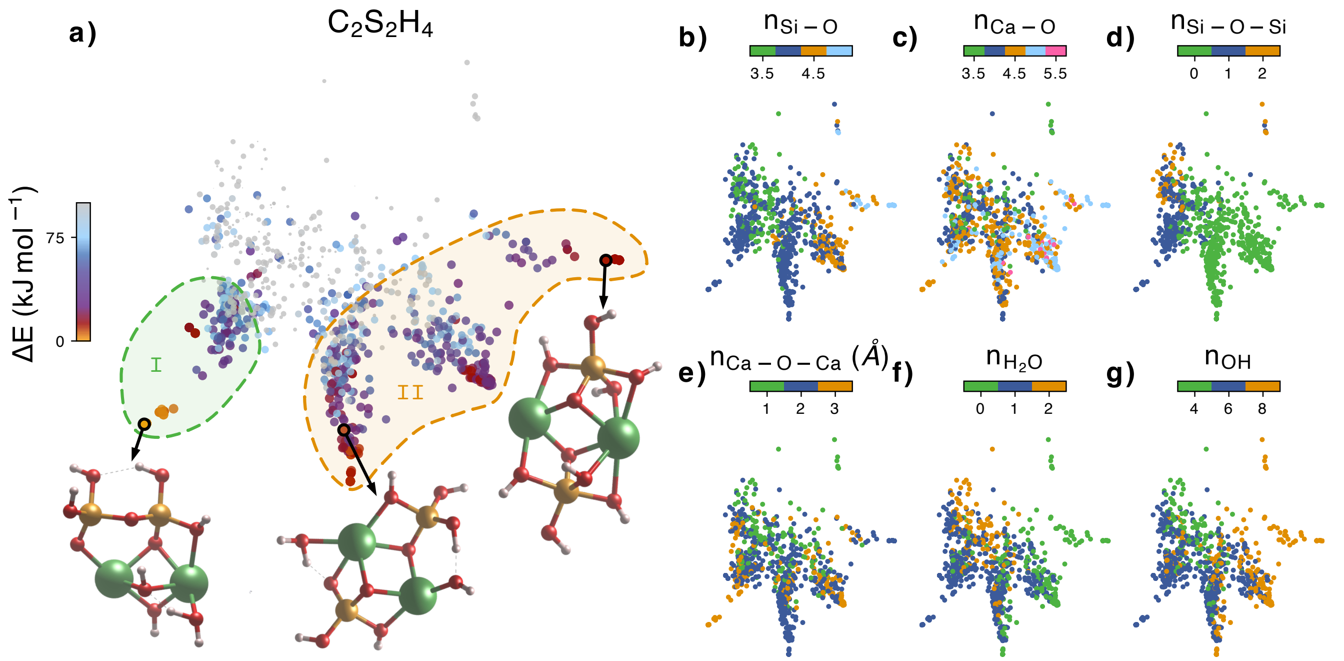

Figure S13. Sketch-map representation of the $C_2S_2H_4$ clusters. The colormap in **(a)** represents the energy difference ΔE. **(b-g)** Collection of structural properties of the $C_2S_2H_4$ clusters mapped into the same Sketch-map. n_{Si-O} refers to the number of Si-O bonds, n_{Ca-O} refers to the number of Ca-O bonds, $n_{Si-O-Sa}$ refers to the number of Si-O-Si bonds, $n_{Ca-O-Ca}$ refers to the Ca-O-Ca bonds, n_{H_2O} refers to the number of water molecules and n_{OH} refers to the number of OH groups. Regions I and II each contain clusters with similar structural properties.

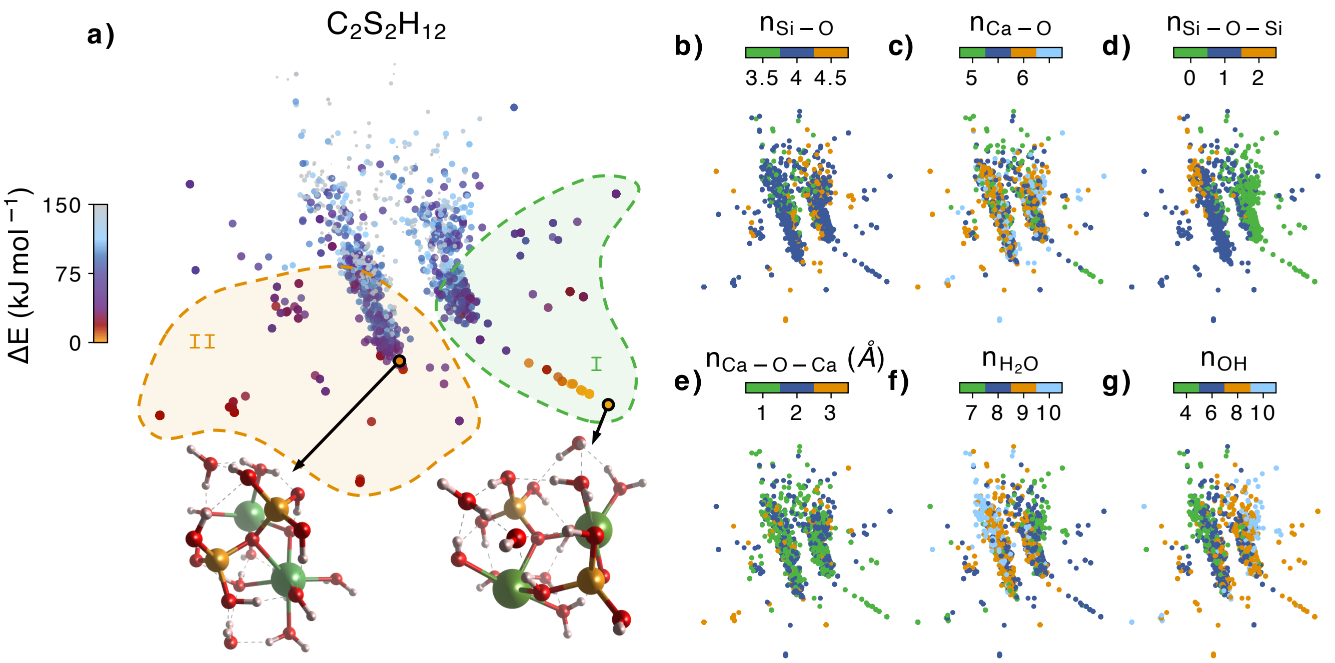

Figure S14. Sketch-map representation of the $C_2S_2H_{12}$ clusters. The colormap in **(a)** represents the energy difference ΔE. **(b-g)** Collection of structural properties of the $C_2S_2H_{12}$ clusters mapped into the same Sketch-map. n_{Si-O} refers to the number of Si-O bonds, n_{Ca-O} refers to the number of Ca-O bonds, $n_{Si-O-Sa}$ refers to the number of Si-O-Si bonds, $n_{Ca-O-Ca}$ refers to the Ca-O-Ca bonds, n_{H_2O} refers to the number of water molecules and n_{OH} refers to the number of OH groups. Regions I and II each contain clusters with similar structural properties.

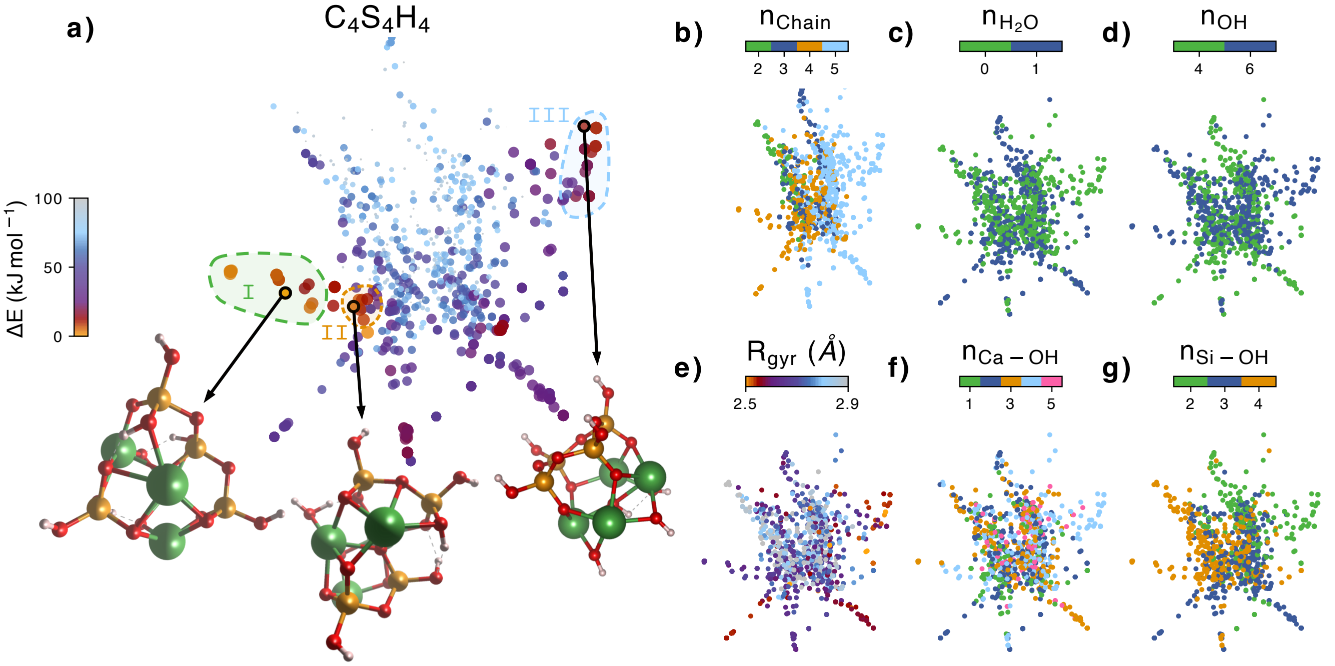

Figure S15. Sketch-map representation of the $C_4S_4H_4$ clusters. The colormap in **(a)** represents the energy difference ΔE. **(b-g)** Collection of structural properties of the $C_4S_4H_4$ clusters mapped into the same Sketch-map. n_{chain} refers to the different silicate chain arrangements defined in Fig. 5 of the main manuscript, n_{H_2O} refers to the number of water molecules, n_{OH} refers to the number of OH groups, R_{gyr} refers to the gyration radius of the clusters, n_{Ca-OH} refers to the number of Ca-OH bonds and n_{Si-OH} refers to the number of Si-OH bonds. Regions I, II and III each contain clusters with similar structural properties.

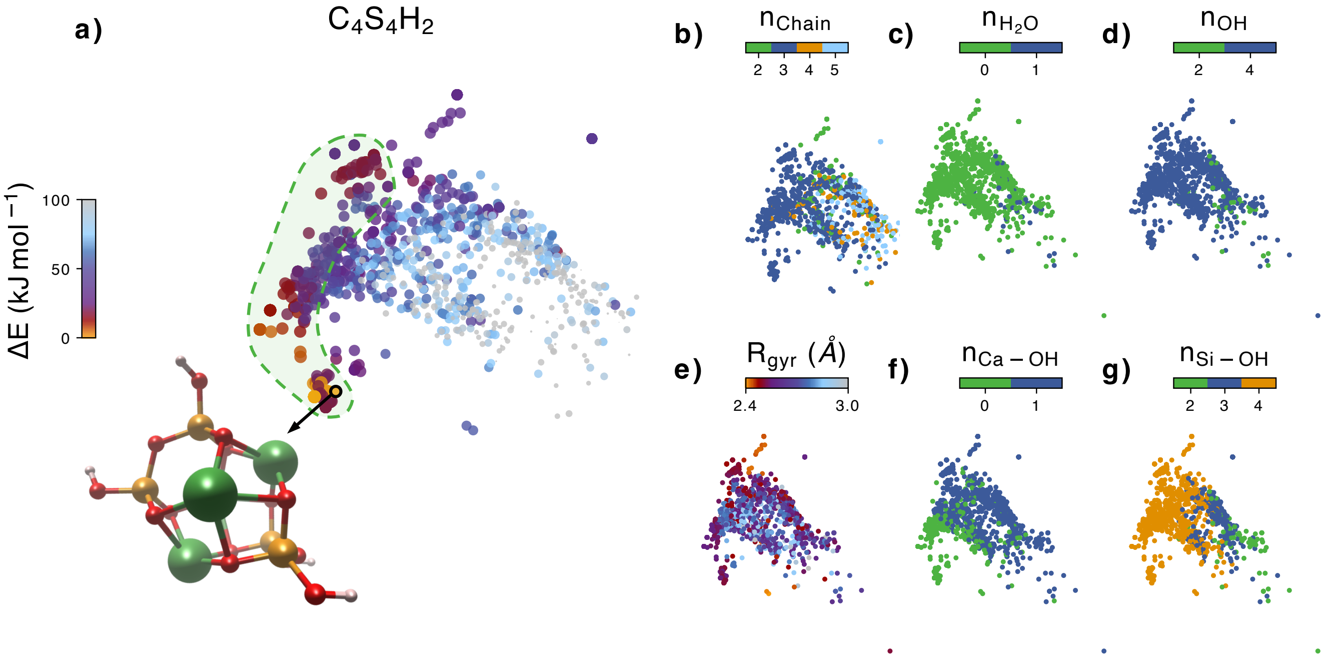

Figure S16. **Sketch-map representation of the $C_4S_4H_2$ clusters**. The colormap in **(a)** represents the energy difference ΔE. **(b-g)** Collection of structural properties of the $C_4S_4H_2$ clusters mapped into the same Sketch-map. n_{chain} refers to the different silicate chain arrangements defined in Fig. 5 of the main manuscript, n_{H_2O} refers to the number of water molecules, n_{OH} refers to the number of OH groups, R_{gyr} refers to the gyration radius of the clusters, n_{Ca-OH} refers to the number of Ca-OH bonds and n_{Si-OH} refers to the number of Si-OH bonds.

Free energies of aggregation

Product	Reagents	ΔG (KJ/mol)
$CSH_{10}-I$	$CaO_2H_3^+ + SiO_5H_5^- + 6H_2O$	-91.07
	$CaO_6H_{12}^{+2} + SiO_9H_{12}^{-2}$ - $2H_2O$	-156,75
$C_2S_2H_{12}-I$	$2CaO_2H_3^+ + 2SiO_5H_5^- + 4H_2O$	-124.13
	$2CaO_6H_{12}^{+2} + 2SiO_9H_{12}^{-2}$ - $12H_2O$	-189.81
	$2CSH_{20}-I$ - $8H_2O$	-66.11
$C_2S_2H_{12}-II$	$2CaO_2H_3^+ + 2SiO_5H_5^- + 4H_2O$	-111.97
	$2CaO_6H_{12}^{+2} + 2SiO_9H_{12}^{-2}$ - $12H_2O$	-177.65
	$2CSH_{10}-I$ - $8H_2O$	-41.80
$C_2S_2H_{20}-I$	$2CaO_2H_3^+ + 2SiO_5H_5^- + 12H_2O$	-127.55
	$2CaO_6H_{12}^{+2} + 2SiO_9H_{12}^{-2}$ - $4H_2O$	-193.23
	2 CSH_{10}	-36.48
$C_2S_2H_{20}-II$	$2CaO_2H_3^+ + 2SiO_5H_5^- + 12H_2O$	-134.23
	$2CaO_6H_{12}^{+2} + 2SiO_9H_{12}^{-2}$ - $4H_2O$	-199.90
	2 CSH_{10}	-43.15
$C_4S_4H_2$	$4CaO_2H_3^+ + 4SiO_5H_5^-$ - $14H_2O$	-140,36
	$4CaO_6H_{12}^{+2} + 4SiO_9H_{12}^{-2}$ - $46H_2O$	-206.04
	$4CSH_{10}-I$ -$38H_2O$	-49.29
	$2CSH_{10}-I + C_2S_2H_{20}-I$ - 38 H_2O	-31.05
	$2C_2S_2H_{20}-I$ - $38H_2O$	-12.81
$C_4S_4H_4$	$4CaO_2H_3^+ + 4SiO_5H_5^-$ - $12H_2O$	-132,01
	$4CaO_6H_{12}^{+2} + 4SiO_9H_{12}^{-2}$ - $44H_2O$	-197.69
	$4CSH_{10}-I$ -$36H_2O$	-40.94
	$2CSH_{10}-I + C_2S_2H_{20}-I$ - 36 H_2O	-22.70
	$2C_2S_2H_{20}-I$ - $36H_2O$	-4.46
$C_4S_4H_8$	$4CaO_2H_3^+ + 4SiO_5H_5^-$ - $8H_2O$	-132,74
	$4CaO_6H_{12}^{+2} + 4SiO_9H_{12}^{-2}$ - $40H_2O$	-198.42
	$4CSH_{10}-I$ -$16H_2O$	-8.61
	$2CSH_{10}-I + C_2S_2H_{20}-I$ - 32 H_2O	-23.42
	$2C_2S_2H_{20}-I$ - $32H_2O$	-5.19

Table S1. **Formation free energies.**

Additional data of cluster aggregation

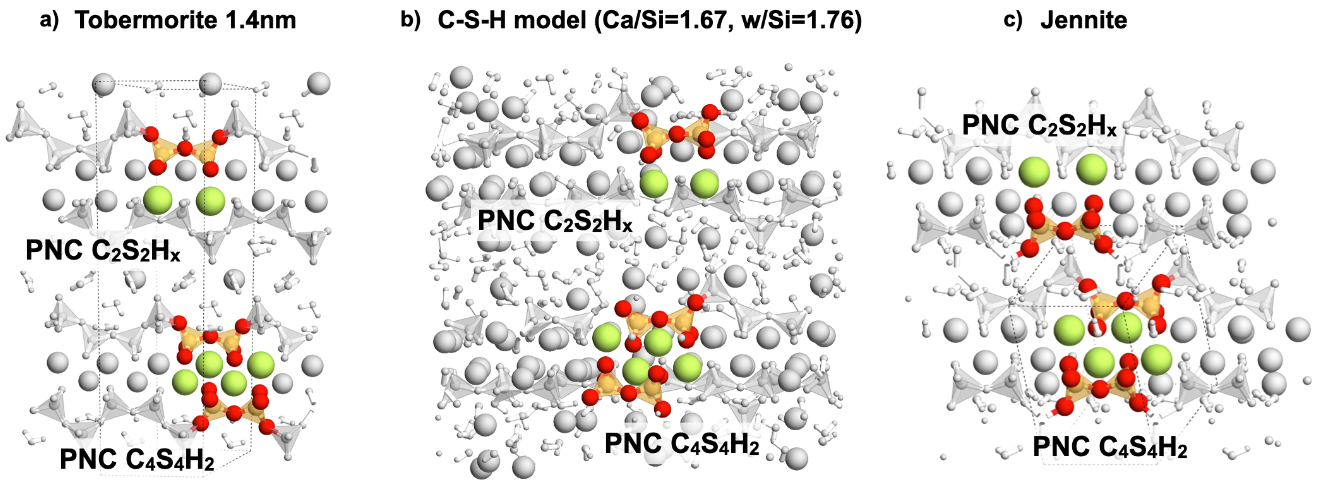

Figure S17. Representation of the atomic structure of **(a)** tobermorite 14, **(b)** C-S-H model and **(c)** Jennite together with the C_2S_2Hx and $C_4S_5H_2$ PPs that act as basic building blocks.

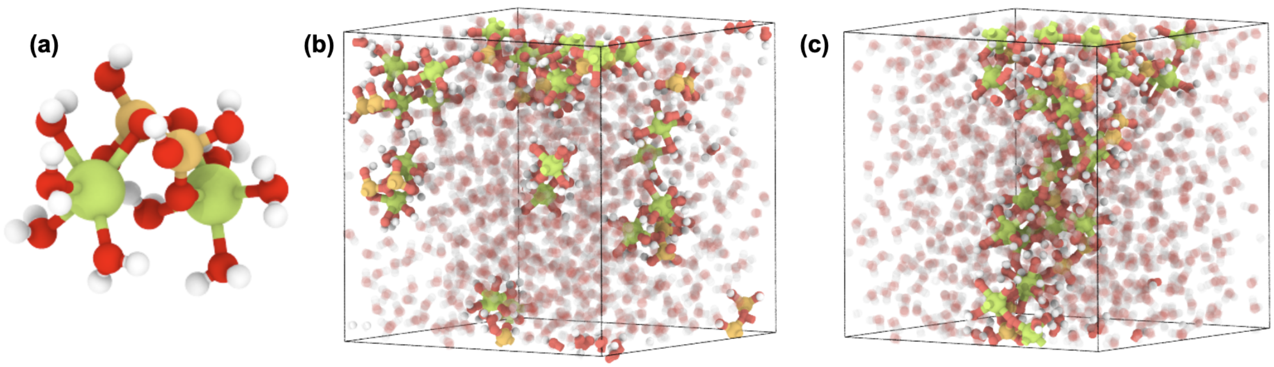

Figure S18. **(a)** Image of the clusters used in the MD simulation, **(b)** the initial random configuration of one of the simulations, **(c)** and the final configuration after the aggregation (15ns).

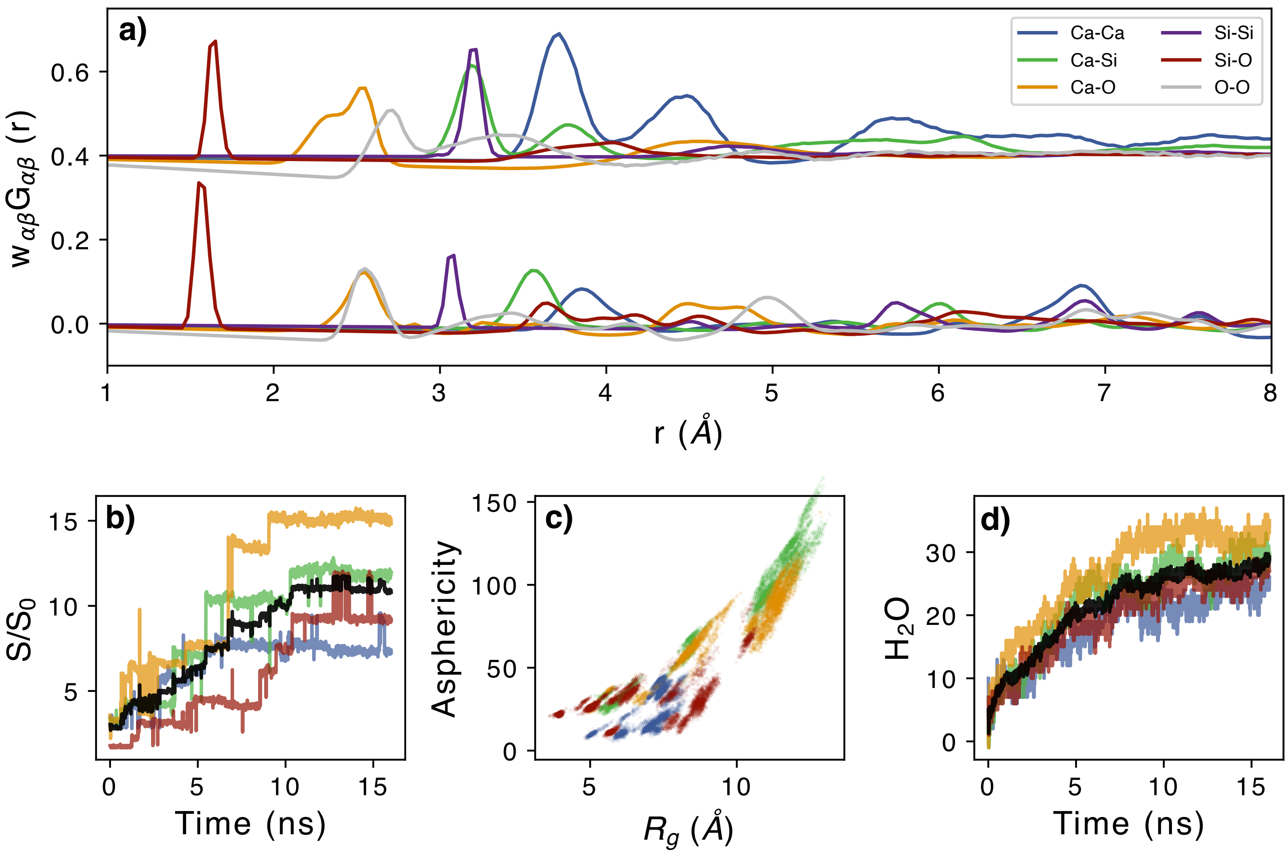

Figure S19. **(a)** Weighted partial pair distribution functions $w_{\alpha\beta}G_{\alpha\beta}$ for the aggregate (top) and tobermorite 14 (bottom). α and β stand for each of the atomic species, and $w_{\alpha\beta}$ is the weight of the partial distribution $G_{\alpha\beta}$ in the total one. This analysis has been helpful to understand the contribution of each atomic pair to the total G(r) and identify the peaks. **(b)** Evolution of the aggregate size S over time for each MD simulation (average in black). The size is defined in terms of number of atoms that for the aggregate normalised by the number of atoms in a primary particle S_0. **(c)** Asphericity of the aggregate as a function of the gyration radius R_g for each MD simulation. **(d)** Water loss $< H_2O >$ from the cluster over time for each MD simulation.

Input Files for the simulation codes

Sample inputs are given in the following files:

- USPEX_input.txt : USPEX input to perform EA. The population size is set to 25 structures and each generation the 60% of structures are used to generate the following generation. From those, 60% are created by the heredity operator, 20% by random generation, 10% by permutation and the remaining 10% by atoms mutation. A 15 minimum distance is set for

- siesta_input.fdf : SIESTA input for the relaxation of the clusters with DFT. GGA-PBE was used as the exchange-correlation functional and double-ζ polarized (DZP) basis set was used for the pseudo atomic orbitals. The Grimme dispersion correction was used to include the van der Walls forces.The convergence criterion for the self-consistent cycle (SCF) was set to 10^{-4} Å. The conjugate gradient (CG) method was used to minimize the positions of the atoms until the maximum force was lest than 0.04 eV/Å.

- gulp_input.in : Input to minimize the structures using the GULP code with the ReaxFF force-field. The ReaxFF parameters are available for download from https://github.com/hegoimanzano/ReaxFF_cement[4] in formated files ready to use with GULP or LAMMPS. The energy was minimized using the NR/BFGS minimiser using a maximum of 50000 steps.

- gaussian_input.com : Gaussian input to compute the free energy of the selected clusters using the Polarizable Continious

Model (PCM). GGA-PBE was used as the exchange-correlation functional using 6-311+G(d) basis set which includes the Grimme dispersion. The dielectric constant value for the PCM was set to $\varepsilon = 56$.

- lammps_input.in : LAMMPS input file for the molecular dynamics simulations with ReaxFF. The charge equilibration method was used, with a cut-off of 10 and a convergence criterion of 10^{-5}. Three simulation stages are done: an energy minization with conjugate gradiend as, a 200 ps for density equilibration in the NPT ensemble, and 6 ns in the NVT ensemble for production. The time step was set to 0.2 fs using the Verlet integrator. The Nose-Hoover thermostat and barostat damping constants were set to 20 and 200 fs respectively.

Supplementary references

1. Eikenberg, J. On the problem of silica solubility at high ph. Tech. Rep., Paul Scherrer Inst.(PSI) (1990).

2. Kutus, B. *et al.* A comprehensive study on the dominant formation of the dissolved ca (oh) 2 (aq) in strongly alkaline solutions saturated by ca (ii). *RSC advances* **6**, 45231–45240 (2016).

3. Šefčík, J. & McCormick, A. V. Thermochemistry of aqueous silicate solution precursors to ceramics. *AIChE J.* **43**, 2773–2784 (1997).

4. Manzano, H. hegoimanzano/reaxff_cement: v01, DOI: 10.5281/zenodo.8379302 (2023).
